# Invasive Pneumococcal Disease 3 Years after Introduction of 10-Valent Pneumococcal Conjugate Vaccine, the Netherlands

**DOI:** 10.3201/eid2111.140780

**Published:** 2015-11

**Authors:** Mirjam J. Knol, Gertjan H.J. Wagenvoort, Elisabeth A.M. Sanders, Karin Elberse, Bart J. Vlaminckx, Hester E. de Melker, Arie van der Ende

**Affiliations:** National Institute for Public Health and the Environment, Bilthoven, the Netherlands (M.J. Knol, E.A.M. Sanders, K. Elberse, H.E. de Melker);; St. Antonius Hospital, Nieuwegein, the Netherlands (G.H.J. Wagenvoort, B.J. Vlaminckx);; University Medical Center Utrecht, Utrecht, the Netherlands (E.A.M. Sanders);; Amsterdam Medical Center, Amsterdam, the Netherlands (A. van der Ende);; Netherlands Reference Laboratory for Bacterial Meningitis, Amsterdam (A. van der Ende)

**Keywords:** invasive pneumococcal disease, IPD, Streptococcus pneumoniae, 10-valent pneumococcal conjugate vaccine, PCV10, 7-valent pneumococcal conjugate vaccine, PCV7, vaccine switching, incidence, surveillance, streptococci, the Netherlands

## Abstract

Three years after a 7-valent pneumococcal conjugate vaccine was replaced by a 10-valent pneumococcal conjugate vaccine in the Netherlands, we observed a decrease in incidence of invasive pneumococcal disease caused by *Streptococcus pneumoniae* serotypes 1, 5, and 7F. Our data do not support or exclude cross-protection against serotype 19A.

A 7-valent pneumococcal conjugate vaccine (PCV7) was first used in the Netherlands in June 2006 in a 3 + 1 schedule for protection against invasive pneumococcal disease (IPD). A switch to 10-valent pneumococcal conjugate vaccine (PCV10) was made in May 2011. There were no catch-up campaigns; children vaccinated with PCV7 completed their series with PCV7. Vaccination coverage has been 94%–95% since PCV7 introduction (*1*).

After PCV7 introduction, vaccine-type IPD incidence decreased for all age groups (*2*). However, nasopharyngeal carriage and incidence of IPD caused by nonvaccine serotypes increased (*2*,*3*). Nevertheless, a 7% decrease in overall IPD incidence was observed 4 years after PCV7 introduction (*2*).

Studies assessing the effect of PCV10 when used in national vaccination programs are limited. Two studies on PCV10 effectiveness in children suggested cross-protection against vaccine-related serotypes, including 19A (*4*,*5*). No studies have been published on herd effects of PCV10 in unvaccinated persons. We report the effect of switching from PCV7 to PCV10 on IPD incidence in the Netherlands.

## The Study

We used data for June 2004–May 2014 from a sentinel laboratory surveillance system, as described (*2*). These data cover ≈25% of the population of the Netherlands. Cumulative incidence ratios (CIRs) with 95% CIs were calculated for comparisons of pre-PCV7 (June 2004–May 2006), pre-PCV10 (June 2009–May 2011), early post-PCV10 (June 2011–May 2013) and 3-year post-PCV10 (June 2013–May 2014) periods. We discerned IPD caused by PCV7 (4, 6B, 9V, 14, 18C, 19F, 23F), non-PCV7, PCV10–7 (present in PCV10 but not PCV7; 1, 5, 7F), non-PCV10, and PCV10-related (6A, 6C, 6D, 7A, 7B, 7C, 9A, 9L, 9N, 18A, 18B, 18F, 19A, 19B, 19C, 23A, 23B) serotypes.

We used nationwide laboratory surveillance data for children <5 years of age available from 2006 to compare IPD incidence rates for a PCV7-eligible cohort (children born March 2008–Feb 2011, >3 months of age, and given a diagnosis of IPD before June 2011) and a PCV10-eligible cohort (children born March 2011–Feb 2014, >3 months of age, and given a diagnosis of IPD before June 2014). Incidence rate ratios (IRRs) with 95% CIs and p values were calculated. Differences between IRRs were tested by calculating p values for interaction between birth cohort and serotype; the IRR for serotypes not related to PCV10 was used as reference.

**Figure 1:**
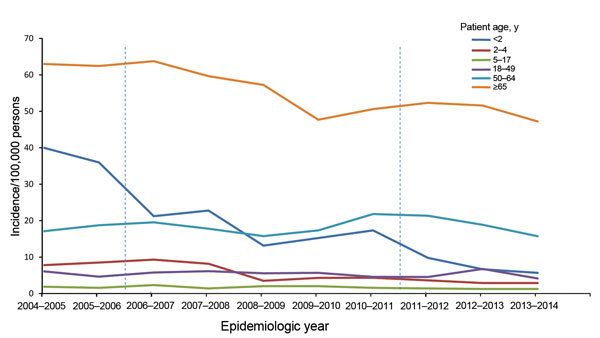
Age-specific incidence of invasive pneumococcal disease caused by any serotype of *Streptococcus pneumoniae* per epidemiologic year (June–May), the Netherlands. Vertical dashed lines indicate introduction of 7-valent pneumococcal conjugate vaccine in June 2006 and 10-valent pneumococcal conjugate vaccine in May 2011. Incidences are based on sentinel surveillance data and extrapolated to the national level.

**Figure 2:**
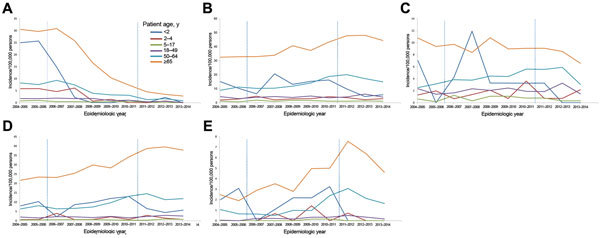
Age-specific incidence of invasive pneumococcal disease caused by different Streptococcus pneumoniae serotypes per epidemiologic year (June–May), the Netherlands. A) 7-valent pneumococcal conjugate vaccine (PCV7) serotypes; B) non-PCV7 serotypes; C) PCV10–7 pneumococcal conjugate vaccine serotypes, D) non-PCV10 serotypes; and E) serotype 19A. Vertical dashed lines indicate introduction of PCV7 in June 2006 and PCV10 in May 2011. Incidences are based on sentinel surveillance data and extrapolated to the national level.

A total of 6,292 IPD cases were included in sentinel surveillance during June 2004–May 2014. By 2009–2011, overall IPD incidence had decreased by 57% for children <2 years of age, 47% for children 2–4 years of age, and 22% for persons >65 years of age (Figure 1; Table 1). No further decrease was observed during 2011–2014 for persons >65 years of age. PCV7 IPD incidence decreased for all age groups, and the decrease continued and showed an overall reduction of 90% by 2013–2014 (Figure 2, panel A). Non-PCV7 IPD incidence increased by 38% for all age groups from 2004–2006 to 2009–2011 (Figure 2, panel B; Table 1).

**Table 1:** Number of IPD cases and cumulative incidence ratios determined on the basis of sentinel surveillance data, the Netherlands, June 2011–May 2013*

Serotype and patient age group, y	No. cases					Comparison, CIR (95% CI)		
Pre-PCV7, 2004–2006	Pre-PCV10, 2009–2011	Early post-PCV10, 2011–2013	3 years post-PCV10, 2013–2014	Pre-PCV10 vs. pre-PCV7	Early post-PCV10 vs. pre-PCV10	3 years post-PCV10 vs. early post-PCV10
All								
<2	75	30	15	5		0.43 (0.28–0.65)	0.51 (0.27–0.94)	0.69 (0.25–1.90)
2–4	25	12	9	4		0.53 (0.27–1.05)	0.75 (0.32–1.79)	0.89 (0.27–2.89)
5–17	22	23	17	8		1.04 (0.58–1.87)	0.75 (0.40–1.40)	0.95 (0.41–2.20)
18–49	197	184	201	73		0.96 (0.78–1.17)	1.10 (0.90–1.34)	0.73 (0.56–0.96)
50–64	273	326	341	134		1.09 (0.93–1.28)	1.03 (0.88–1.19)	0.78 (0.64–0.95)
>65	717	622	703	338		0.78 (0.70–0.87)	1.06 (0.95–1.18)	0.91 (0.80–1.03)
Total	1,309	1,197	1,286	562		0.90 (0.83–0.97)	1.06 (0.98–1.15)	0.87 (0.79–0.96)
PCV7								
<2	50	1	2	0		0.02 (0.00–0.16)	2.03 (0.18–22.33)	NC
2–4	18	2	1	0		0.12 (0.03–0.53)	0.50 (0.05–5.55)	NC
5–17	11	6	2	1		0.54 (0.20–1.47)	0.34 (0.07–1.67)	1.01 (0.09–11.1)
18–49	63	35	17	2		0.57 (0.38–0.86)	0.49 (0.27–0.87)	0.24 (0.05–1.03)
50–64	120	53	25	7		0.40 (0.29–0.56)	0.46 (0.29–0.74)	0.56 (0.24–1.29)
>65	344	112	54	20		0.29 (0.24–0.36)	0.45 (0.33–0.62)	0.70 (0.42–1.17)
Total	606	209	101	30		0.34 (0.29–0.40)	0.48 (0.38–0.61)	0.59 (0.39–0.89)
Non-PCV7								
<2	25	29	13	5		1.24 (0.73–2.12)	0.45 (0.24–0.87)	0.80 (0.28–2.23)
2–4	7	10	8	4		1.57 (0.60–4.13)	0.80 (0.32–2.04)	1.00 (0.30–3.32)
5–17	11	17	15	7		1.54 (0.72–3.30)	0.89 (0.44–1.78)	0.94 (0.38–2.31)
18–49	134	149	184	71		1.14 (0.90–1.44)	1.24 (1.00–1.54)	0.78 (0.59–1.02)
50–64	153	273	316	127		1.63 (1.34–1.99)	1.14 (0.97–1.33)	0.80 (0.65–0.98)
>65	373	510	649	318		1.24 (1.08–1.41)	1.19 (1.06–1.34)	0.93 (0.81–1.06)
Total	703	988	1,185	532		1.38 (1.26–1.52)	1.19 (1.09–1.29)	0.89 (0.81–0.99)
PCV10–7								
<2	7	6	3	0		0.92 (0.31–2.73)	0.51 (0.13–2.02)	NC
2–4	5	6	2	3		1.32 (0.40–4.33)	0.34 (0.07–1.66)	3.00 (0.50–18.0)
5–17	5	12	7	2		2.40 (0.84–6.81)	0.59 (0.23–1.49)	0.58 (0.12–2.77)
18–49	69	78	91	26		1.16 (0.84–1.60)	1.18 (0.87–1.59)	0.58 (0.37–0.89)
50–64	43	83	97	26		1.77 (1.22–2.55)	1.15 (0.86–1.54)	0.53 (0.35–0.82)
>65	115	114	119	47		0.90 (0.69–1.16)	0.98 (0.76–1.26)	0.75 (0.53–1.05)
Total	244	299	319	104		1.21 (1.02–1.43)	1.06 (0.90–1.24)	0.65 (0.52–0.81)
Non-PCV10								
<2	18	23	10	5		NA	0.44 (0.21–0.92)	1.03 (0.35–3.03)
2–4	2	4	6	1		NA	1.51 (0.43–5.35)	0.33 (0.04–2.77)
5–17	6	5	8	5		NA	1.61 (0.53–4.93)	1.26 (0.41–3.85)
18–49	65	71	93	45		NA	1.32 (0.97–1.80)	0.98 (0.68–1.39)
50–64	110	190	219	101		NA	1.13 (0.93–1.37)	0.92 (0.72–1.16)
>65	258	396	530	271		NA	1.25 (1.10–1.43)	0.97 (0.84–1.12)
Total	459	689	866	428		NA	1.25 (1.13–1.38)	0.98 (0.88–1.11)
19A								
<2	5	5	0	0		1.07 (0.31–3.70)	0.00 (NC)	NC
2–4	0	2	1	0		NC	0.50 (0.05–5.55)	NC
5–17	0	2	0	0		NC	0.00 (NC)	NC
18–49	1	12	15	3		12.3 (1.60–94.6)	1.26 (0.59–2.69)	0.40 (0.12–1.39)
50–64	13	28	44	14		1.97 (1.02–3.81)	1.54 (0.96–2.48)	0.63 (0.35–1.15)
>65	25	63	94	33		2.28 (1.43–3.62)	1.40 (1.02–1.92)	0.66 (0.45–0.99)
Total	44	112	154	50		2.50 (1.77–3.55)	1.36 (1.07–1.74)	0.65 (0.47–0.89)

*PCV7, 7-valent pneumococcal conjugate vaccine; PCV10−7, serotypes present in PCV10 but not PCV7 (3 additional serotypes); PCV10, 10-valent pneumococcal conjugate vaccine; CIR, cumulative incidence ratio; NC, not calculated (numbers too low to be informative or relevant). NA, not applicable.

Overall PCV10–7 IPD incidence increased slightly after PCV7 introduction (CIR 1.21, 95% CI 1.02–1.43) (Figure 2 panel C; Table 1). For children <2 years of age, PCV10–7 IPD incidence decreased 2 years after the switch to PCV10, although not significantly, because of a low number of cases (CIR 0.51, 95% CI 0.13–2.02). In the third year (2013–2014) after PCV10 introduction, no IPD cases were caused by serotypes 1, 5, and 7F in children <2 years of age. For other age groups, PCV10–7 IPD incidence did not change in the 2 years after PCV10 introduction, but in the third year, incidence decreased by 42% and 47% for persons 18–49 years of age and 50–64 years of age, respectively; a 25% decrease was observed for persons >65 years of age (Figure 2 panel C; Table 1). Non-PCV10 IPD incidence increased for most age groups in the 2 years after PCV10 introduction (overall CIR 1.25, 95% CI 1.13–1.38) but did not increase further in 2013–2014 (Figure 2, panel D; Table 1), partly because of a decrease in 19A IPD (Figure 2, panel E; Table 1).

The IPD incidence rate for the PCV10-eligible cohort was lower than that for the PCV7-eligible cohort for PCV10–7 serotypes (IRR 0.04, 95% CI 0.01–0.27), PCV7-related serotypes (IRR 0.38, 95% CI 0.19–0.77), and non-PCV10 serotypes (IRR 0.67, 95% CI 0.46–0.99) (Table 2). The decrease in PCV10–7 IPD was greater than that for non-PCV10 serotypes (p_interaction_ = 0.005). However, IRRs for PCV10-related IPD and specifically serotype 19A were not different from the IRR for PCV10-unrelated IPD (p_interaction_ = 0.229/0.165).

**Table 2:** Number of IPD cases and incidence rate ratios determined on the basis of sentinel surveillance data, the Netherlands, June 2011–May 2103*

Variable	PCV7-eligible cohort	PCV10-eligible cohort	IRR (95% CI)†	Exact p value	p value for interaction‡
Birth cohort	2008 Mar 1–2011 Feb 28	2011 Mar 1–2014 Feb 28	NA	NA	NA
Observation period	2008 Jun 1–2011 May 31	2011 Jun 1–2014 May 31	NA	NA	NA
Persons at risk	550,297	537,071	NA	NA	NA
Person-years at risk	822,100	814,980	NA	NA	NA
No. (%) IPD cases per 100,000 persons					
Serotypes					
PCV7§	5 (0.6)	0 (0.0)	NC	0.063	NC
6B	2	0	NC	NC	NC
18C	2	0	NC	NC	NC
19F	1	0	NC	NC	NC
PCV10–7	27 (3.3)	1 (0.1)	0.04 (0.01–0.27)	<0.001	0.005
1	2	0	NC	NC	NC
5	2	0	NC	NC	NC
7F	23	1	0.04 (0.01–0.32)	<0.001	0.009
PCV10-related§	33 (4.0)	14 (1.7)	0.43 (0.23–0.80)	0.006	0.229
6A	2	0	NC	NC	NC
6C	0	1	NC	NC	NC
9N	0	1	NC	NC	NC
19A	29	11	0.38 (0.19–0.77)	0.005	0.165
23A	1	1	NC	NC	NC
23B	1	0	NC	NC	NC
PCV10-nonrelated	63 (7.7)	42 (5.2)	0.67 (0.46–0.99)	0.045	Reference
3	4	0	NC	NC	NC
8	3	2	NC	NC	NC
10A	14	16	NC	NC	NC
11A	3	0	NC	NC	NC
12F	2	2	NC	NC	NC
15A	1	0	NC	NC	NC
15B	3	1	NC	NC	NC
15C	1	4	NC	NC	NC
16F	4	1	NC	NC	NC
17F	3	1	NC	NC	NC
22F	5	3	NC	NC	NC
24B	0	1	NC	NC	NC
24F	3	1	NC	NC	NC
27	2	1	NC	NC	NC
33F	11	6	NC	NC	NC
34	1	0	NC	NC	NC
35B	0	1	NC	NC	NC
35F	2	1	NC	NC	NC
38	1	1	NC	NC	NC
Total	128 (15.6)	57 (7.0)	0.45 (0.33–0.61)	<0.001	NA

*IPD, invasive pneumococcal disease; PCV7, 7-valent pneumococcal conjugate vaccine; PCV10−7, serotypes present in PCV10 but not PCV7 (3 additional serotypes); PCV10, 10-valent pneumococcal conjugate vaccine; IRR, incidence rate ratio; NA, not applicable; NC, not calculated (numbers too low to be informative or relevant). †PCV10-eligible cohort vs. PCV7-eligible cohort.  ‡For difference between IRRs. §serotypes with no IPD cases in both cohorts are not shown.

## Conclusions

We observed a decrease in PCV7-type IPD >8 years after PCV7 introduction for all age groups. However, this decrease was lessened by an increase in non–vaccine-type IPD, a finding similar to that reported in other countries (*6*,*7*). There was an overall 80% decrease in IPD incidence for children <5 years of age and a 25% decrease for persons >65 years of age.

PCV10 introduction caused a decrease in PCV10–7 IPD incidence in PCV10-eligible children, providing evidence for a direct effect from PCV10. Potential cross-protection of PCV10 against serotype 19A, as corroborated by a case–control study showing 82% effectiveness against 19A IPD (*5*), is still debated. In our study, the incidence rate for 19A IPD was lower in the PCV10-eligible cohort than the PCV7-eligible cohort, but the decrease in 19A IPD was not different from the decrease in PCV10-unrelated IPD, which precludes drawing conclusions about cross-protection against 19A IPD. In addition, 19A carriage had already decreased in toddlers before PCV10 introduction (*8*).

We observed a decrease in non–PCV10 IPD in the PCV10-eligible cohort but have no indication that surveillance sensitivity changed over time. The decrease might be caused by natural fluctuations or different viral seasons (*9*). A study in Canada reported lower incidence rates for 19A IPD and other non–vaccine-type IPD in a PCV10-eligible cohort (*4*). It was hypothesized that lower antibody levels induced by PCV10 (*10*,*11*) might lead to smaller disturbances of the nasopharyngeal niche and replacement by new serotypes against which there is no immunity, which might result in a lower incidence of non-PCV10 IPD. However, a randomized controlled trial showed similar carriage rates for non-PCV10 serotypes, including 19A, for infants vaccinated with PCV7 and those vaccinated with PCV10 (*12*).

In the third year after PCV10 introduction, PCV10–7 IPD incidence also decreased in nonvaccinated age groups, which might indicate herd effects. After PCV7 introduction, herd effects appeared after 3 years (*13*). Non-PCV10 IPD incidence did not increase in the second and third years after PCV10 introduction, which was partially caused by a reduction in 19A IPD. Longer follow-up times are needed to distinguish whether these observations were caused by cross-protection against 19A in children through herd effects of PCV10, reduced nonvaccine serotype replacement by PCV10, or temporal fluctuations.

We used data from a stable surveillance system with constant coverage over time; age and serotype data were nearly complete (99.9%). However, a limitation of our study was the ecologic design. Thus, one should be cautious in interpreting findings as causally related to vaccination. Also, we have limited data on IPD before PCV7 introduction and after the switch to PCV10.

In conclusion, PCV10 introduction in 2011 decreased vaccine-type IPD incidence in targeted birth cohorts. Three years after introduction, herd effects became apparent. Stabilization of non-PCV10 IPD in the second and third years after PCV10 introduction might indicate reduced serotype replacement by PCV10 or cross-protection against 19A. However, we cannot make firm conclusions on cross-protection of PCV10 against serotype 19A. Continued surveillance of serotype-specific IPD is crucial for evaluating long-term effects of pneumococcal conjugate vaccines in human populations.
