# Correction: Vol. 21, No. 11

**DOI:** 10.3201/eid2201.C12201

**Published:** 2016-01

**Keywords:** errata, erratum, correction

Details regarding vaccine serotypes and surveillance programs were described incorrectly in Invasive Pneumococcal Disease 3 Years after Introduction of 10-Valent Pneumococcal Conjugate Vaccine, the Netherlands (M.J. Knol al.). The article has been corrected online (http://wwwnc.cdc.gov/eid/article/21/11/14-0780_article).

